# The effect of SMAD4 on the prognosis and immune response in hypopharyngeal carcinoma

**DOI:** 10.3389/fmed.2023.1139203

**Published:** 2023-03-23

**Authors:** Juanjuan Song, Jialing Wu, Jiaxuan Ding, Yangxin Liang, Changlong Chen, Yimin Liu

**Affiliations:** Department of Radiation Oncology, Sun Yat-sen Memorial Hospital, Sun Yat-sen University, Guangzhou, China

**Keywords:** head and neck cancer, SMAD4, CD8+ cytotoxic T cell, CD15+ neutrophils, immune response

## Abstract

**Objectives:**

In malignant tumors, elevated infiltration of intratumoral CD8+ cytotoxic T cells predicts a beneficial prognosis, whereas high levels of CD15+ neutrophils in peritumor tissues indicate poor prognosis. It is unclear how SMAD4, which promotes favorable clinical outcomes and antitumor immunoregulation, along with CD8+ cytotoxic T cells and CD15+ neutrophils exert an influence on hypopharyngeal carcinoma (HPC).

**Materials and methods:**

Specimens were collected from 97 patients with HPC. Immunohistological analyses of SMAD4, CD8+ cytotoxic T cell and CD15+ neutrophil expression were performed. SMAD4 nuclear intensity was measured, meanwhile, CD8+ cytotoxic T cells and CD15+ neutrophils were counted under a microscope. The prognostic role of SMAD4 was determined using the log-rank test and univariate and multivariate analyses. The relationship among SMAD4, CD8+ cytotoxic T cells, and CD15+ neutrophils was estimated by Mann–Whitney U test.

**Results:**

High levels of SMAD4 were associated with favorable overall survival (OS) and disease-free survival (DFS) in HPC. Multivariate analysis suggested that SMAD4 is an independent predictor of OS and DFS. A high density of intratumoral CD8+ cytotoxic T cells and low accumulation of CD15+ neutrophils in the peritumor area were associated with longer OS and DFS. Furthermore, SMAD4 was linked to the levels of intratumoral CD8+ cytotoxic T cells and peritumoral CD15+ neutrophils. Patients with high SMAD4/high intratumoral CD8+ cytotoxic T cells or high SMAD4/low peritumoral CD15+ neutrophils showed the best prognosis.

**Conclusion:**

SMAD4, CD8+ cytotoxic T cell level, and CD15+ neutrophil level have prognostic value in HPC. SMAD4 is a promising prognostic marker reflecting immune response in HPC.

## Introduction

Hypopharyngeal carcinoma (HPC) is one of the most lethal head and neck squamous cancers, with a 5-year overall survival (OS) rate of 30–35% (1, 2). According to the Global Cancer Statistics 2020, newly diagnosed cancer cases and the hypopharynx-related death rate were 84,254 and 38,599, respectively, with the incidence and mortality in males accounting for 83.4% and 83.7% (3), respectively. Most cases of HPC are diagnosed in advanced stages (stage III or IV), as the disease presents with few specific or evident symptoms in early stages because of its anatomical location (4). Despite recent advances in treatment modalities consisting of surgery, chemotherapy, and radiotherapy, the disease prognosis is far from satisfactory (5). Additionally, total laryngectomy decreases the quality of life of patients suffering from HPC. Hence, a novel approach for forecasting prognosis of HPC is desperately needed.

SMAD4, also known as DPC4, was initially identified in pancreatic carcinoma at chromosome18q21.1 (6). Composed of three main domains, Mad homology 1, Mad homology 2, and an interposed linker region (7), SMAD4 mostly accumulates in the nucleus (8). SMAD4 is a pivotal transducer in the transforming growth factor-beta (TGF-β) signaling pathway and has been suggested to exert anti-tumor effects by regulating cell proliferation, differentiation, migration, and apoptosis (9). Mutation or inactivation of SMAD4 promotes tumor metastasis, recurrence, or drug resistance, including in colorectal cancer (10), pancreatic ductal carcinoma (11), and lung cancer (12).

The tumor microenvironment comprises tumor cells, stromal cells, epithelial cells, fibroblasts, adipocytes, and immune cells (T cells, B cells, neutrophils, and macrophages) (13, 14). Notably, tumor-infiltrating lymphocytes play a critical role in anti-tumor response (15), and densely aggregated CD8+ cytotoxic T cells indicate a favorable prognosis as the dominant effector immune cells (16). Tumor-associated neutrophils polarize into two phenotypes: anti-tumoral and pro-tumoral (17). For example, CD15, also known as fucosyltransferase 4, LeuM1, Lewis X, or stage-specific embryonic antigen 1 (18), is a differentiation marker expressed on neutrophils, which suppresses immune responses and contributes to angiogenesis and tumor progression (19). SMAD4 participates in immune processes by influencing infiltrating immune cells. For instance, SMAD4-deficient T cells exhibit poor proliferation, highlighting the role of SMAD4 in this process (20).

Although SMAD4 plays a crucial role in tumor prognosis and antitumor immunoregulation, its importance in the prognosis and immune response in HPC remains unclear. Therefore, we investigated the expression of SMAD4 in HPC tissues to evaluate its prognostic value. We also counted intratumoral CD8+ T cells and peritumoral CD15 + neutrophils and analyzed their relationship with SMAD4 level to determine the effect of SMAD4 on immune response in HPC.

## Materials and methods

### Patients

This study was approved by the Ethics Committee of Sun Yat-sen Memorial Hospital, which granted a waiver of informed consent to use patient records and pathological samples for research. Patients were excluded if they had an initial definitive experience with chemoradiotherapy, lacked adequate follow-up information, or were denied medical treatment. Overall, we selected and analyzed 97 eligible patients who were diagnosed with squamous cell carcinoma of hypopharynx. All patients underwent surgery with or without adjuvant therapy, and the Tumor Node Metastasis stage was determined according to the guidelines stated by the American Joint Committee on Cancer for hypopharyngeal carcinoma (7th ed.).

### Specimens and immunohistochemistry

Paraffin-embedded specimens collected from surgically resected tumors were prepared for immunohistochemistry staining based on standard protocols as follows. The tissue sections were incubated at 60°C for 1.5 h. All slides were treated with dimethylbenzene for de-paraffinization and rehydrated in a graded ethanol series. The slides were washed with phosphate-buffered saline (PBS, 3×), and then immersed in EDTA (pH 9.0) and baked at high heat for 10 min, followed by medium heat for 15 min for antigen-retrieval. The sections were cooled and then incubated in hydrogen peroxide to block endogenous peroxidase activity. After 10 min, the slides were washed with PBS (3×); incubated in goat serum for 30 min; and incubated with a primary rabbit monoclonal anti-SMAD4 antibody (1:200 dilution, Abcam, Cambridge, UK), primary rabbit monoclonal anti-CD8 antibody (1:100 dilution, Zhongshan Golden Bridge Biotech, Beijing, China), or primary rabbit monoclonal anti-CD15 antibody (1:200 dilution, Zhongshan Golden Bridge Biotech) overnight at 4°C. The slides were warmed to room temperature for 30 min and washed with PBS (5×). The sections were incubated with a horseradish peroxidase-conjugated secondary antibody (Envision Detection Kit, GK500705, Agilent Technologies, Santa Clara, CA, USA) for 30 min, followed by incubation in 3,30-diaminobenzidine tetrahydrochloride for visualization. The reactions were terminated by rinsing with running water. Hematoxylin was used to stain the sections for 4 min. After washing, the slides were treated with 1% hydrochloric acid alcohol for 5 s and sealed with neutral resins.

### Evaluation

Immunohistochemistry staining of SMAD4 was assessed on a scale of 0–3 by two researchers. A score of 0 indicated negative SMAD4 expression, 1 indicated slight expression, 2 indicted medium expression, and 3 indicated strong expression. Scores of 0 and 1 were further categorized as low expression and 2 and 3 were categorized as high expression. The total CD8+ cytotoxic T cell and CD15+ neutrophil counts were manually calculated under a microscope in five random fields of view.

### Statistical analysis

All statistical analyses were performed with GraphPad Prism 8.4.3 software (GraphPad, Inc., La Jolla, CA, USA) and SPSS version 25.0 software (SPSS, Inc., Chicago, IL, USA). Fisher’s exact test or Chi-square test were used to compare clinical features. Mann–Whitney U test was used to investigate the relevance between SMAD4 and CD8+ cytotoxic T cells or CD15+ neutrophils. The Kaplan–Meier method and log-rank test were applied to reveal the survival curves. Univariate and multivariate analyses were conducted with Cox proportional hazards model to evaluate the independent risk factors correlated with overall survival (OS) and disease-free survival (DFS). A *P*-value ≤0.05 was considered statistically significant.

## Results

### Clinicopathologic features of patients with HPC

The clinicopathologic parameters of the 97 patients with HPC are shown in Table 1. Consistent with the global epidemiology, HPC cases were predominantly male, accounting for roughly 97% of patients, with a median age of 59 years (range, 40–74 years). HPC is mainly diagnosed during late stages and thus only one case of stage I and II were included in our data. Twenty-three patients (24%) were diagnosed with T1–2 and 74 patients (76%) were T3–4. Additionally, 35 patients (36%) were confirmed as N0–1 and 62 patients (64%) as N2–3. Well-differentiated and poorly differentiated tumors accounted for 82% and 18% of cases, respectively, according to the histological grade. Only eight patients showed positive surgical margins. Over half of the patients (80%) showed no signs of vascular invasion, whereas 20% of patients showed vessels with varying degrees of carcinoma invasion. Moreover, adjuvant chemotherapy was administered to 58 patients, and almost all patients (96) were treated with adjuvant radiotherapy. The proportion of patients have local recurrence and distant metastasis was 34% and 23%, respectively.

**TABLE 1:** The relationship between SMAD4 expression and clinical characteristics of HPC patients.

Variables	High SMAD4 expression (*n* = 66)	Low SMAD4 expression (*n* = 31)	*P*-value
**Age (years)**			
<59	37	10	0.0287*
≥59	29	21	
**Gender**			
Male	64	30	>0.9999
Female	2	1	
**TNM stage**			
I–II	1	0	>0.9999
III–IV	65	31	
**Tumor differentiation**			
Well differentiated	53	27	0.5694
Poor differentiated	13	4	
**Surgical margin**			
Positive	6	2	>0.9999
Negative	60	29	
**Vascular invasion**			
Yes	15	4	0.2893
No	51	27	
**Adjuvant chemotherapy**			
Yes	39	19	0.8368
No	27	12	
**Adjuvant radiotherapy**			
Yes	65	31	> 0.9999
No	1	0	
**T stage**			
1–2	16	7	0.8576
3–4	50	24	
**N stage**			
0–1	24	11	0.9329
2–3	42	20	
**Local recurrence**			
Yes	16	17	0.0030*
No	50	14	
**Distant metastasis**			
Yes	9	13	0.0019*
No	57	18	

*Means statistically significant.

### Characteristics and evaluation of SMAD4 expression in HPC

**FIGURE 1:**
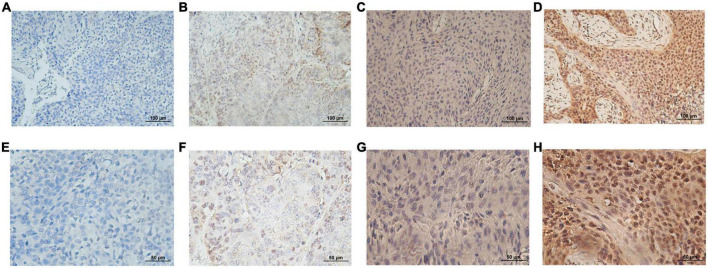
Representative immunohistological staining for each SMAD4 score. Low expression level of SMAD4 was defined by scores of 0 **(A,E)** and 1 **(B,F)** and high expression level of SMAD4 was defined by scores of 2 **(C,G)** and 3 **(D,H)** (upper panel, ×200 magnification; lower panel, ×400 magnification).

SMAD4 expression level was evaluated by immunohistochemistry analysis, which revealed a variable degree of nuclear staining intensity. Representative images are shown in Figures 1A–H. Overall, 31 HPC samples showed reduced levels of SMAD4 (32%), whereas 68% of specimens were in the SMAD4-sufficient group (*n* = 66). The association between SMAD4 expression and the patients’ clinicopathologic characteristics is summarized in Table 1. SMAD4 expression was associated with age (*P* = 0.0287), local recurrence (*P* = 0.0030) and distant metastasis (*P* = 0.0019). Nevertheless, the status of SMAD4 did not significantly differ by sex (*P* > 0.9999), Tumor Node Metastasis stage (*P* > 0.9999), T stage (*P* = 0.8576), N stage (*P* = 0.9329), tumor differentiation (*P* = 0.5694), surgical margin (*P* > 0.9999), vascular invasion (*P* = 0.2983), adjuvant chemotherapy (*P* = 0.8368), or adjuvant radiotherapy (*P* > 0.9999).

### Association between SMAD4 expression and patient prognosis

**FIGURE 2:**
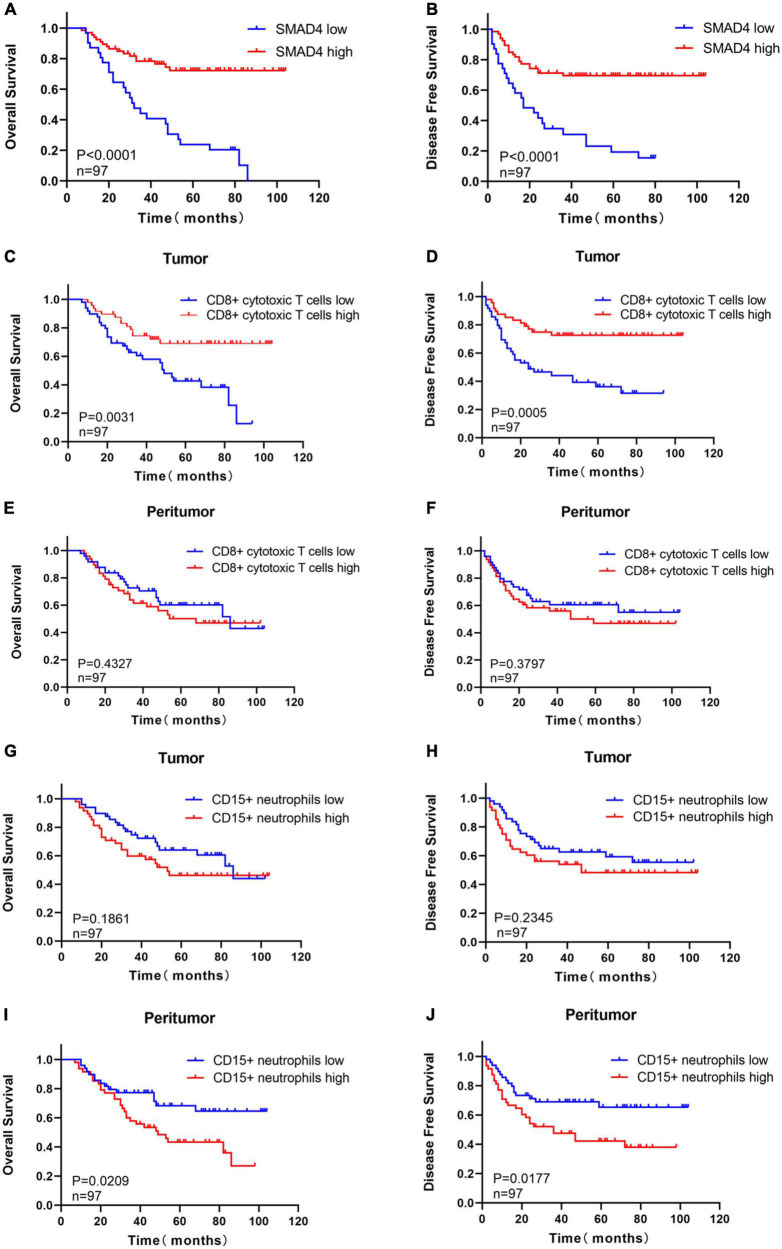
Prognostic value of SMAD4, CD8 + cytotoxic T cells, and CD15 + neutrophils in HPC. High expression level of SMAD4 indicates longer OS **(A)** and DFS **(B)**. Prognostic value of intratumoral **(C,D)** or peritumoral **(E,F)** CD8 + cytotoxic T cells in HPC. The prognostic value of intratumoral **(G,H)** or peritumoral **(I,J)** CD15 + neutrophils in HPC. DFS, disease-free survival; HPC, hypopharyngeal carcinoma; OS, overall survival.

The prognostic value of SMAD4 expression was estimated by comparing OS and DFS in HPC patients. The median OS and DFS were 49 and 47 months, respectively, for HPC patients with high SMAD4 expression level, while it was 31 and 17 months, respectively, for patients with low SMAD4 expression level. Moreover, Kaplan–Meier analysis explicitly revealed that high levels of SMAD4 expression improved OS (Figure 2A) and DFS (Figure 2B), whereas SMAD4 deficits contributed to undesirable clinical outcomes. Univariate and multivariate analyses confirmed that the SMAD4 abundance strongly influenced the OS and DFS (Tables 2, 3), suggesting that SMAD4 has prognostic value in patients with HPC.

**TABLE 2:** Univariate and multivariate analyses of variables associated with overall survival.

Variables	Univariate analysis			Multivariate analysis		
	HR	95% CI	*P*	HR	95% CI	*P*
**Age (year)**						
≥59 vs. <59	1.512	0.824–2.775	0.182			
**Gender**						
Female vs. male	0.046	0.000–25.593	0.34			
**TNM stage**						
III + IV vs. I + II	20.578	0.000–2,068,556.499	0.607			
**T stage**						
3–4 vs. 1–2	1.193	0.570–2.496	0.639			
**N stage**						
2–3 vs. 0–1	1.744	0.895–3.399	0.102			
**Tumor differentiation**						
Poorly vs. well	1.101	0.510–2.375	0.807			
**Surgical margin**						
Negative vs. positive	0.752	0.268–2.105	0.587			
**Vascular invasion**						
No vs yes	0.601	0.295–1.224	0.161			
**Adjuvant chemotherapy**						
No vs. yes	0.808	0.437–1.496	0.498			
**Adjuvant radiotherapy**						
No vs. yes	0.049	0.000–4884.866	0.607			
**CD8 + cytotoxic T cells expression (tumor)**						
Low vs. high	2.547	1.336–4.855	0.005*	1.021	0.429–2.431	0.962
**CD8 + cytotoxic T cells expression (Peritumor)**						
Low vs. high	0.788	0.432–1.435	0.436			
**CD15 + neutrophils expression (tumor)**						
Low vs. high	0.669	0.366–1.222	0.191			
**CD15 + neutrophils expression (peritumor)**						
Low vs. high	0.486	0.259–0.911	0.024*	0.668	0.350–1.276	0.222
**SMAD4 expression**						
Low vs high	4.42	2.384–8.195	<0.0001*	3.95	1.691–9.225	0.002*

*Means statistically significant.

**TABLE 3:** Univariate and multivariate analyses of variables associated with disease-free survival.

Variables	Univariate analysis			Multivariate analysis		
	HR	95% CI	*P*	HR	95% CI	*P*
**Age (year)**						
≥ 59 vs <59	1.729	0.941–3.177	0.078			
**Gender**						
Female vs. male	0.046	0.000–31.050	0.355			
**TNM stage**						
III + IV vs. I + II	20.557	0.000–1,688,499.375	0.601			
**T stage**						
3–4 vs. 1–2	1.349	0.648–2.809	0.424			
**N stage**						
2–3 vs. 0–1	2.070	1.045–4.100	0.037*	1.732	0.843–3.558	0.135
**Tumor differentiation**						
Poorly vs. well	1.041	0.484–2.240	0.918			
**Surgical margin**						
Negative vs. positive	0.819	0293–2.290	0.703			
**Vascular invasion**						
No vs. yes	0.392	0.204–0.754	0.005*	0.280	0.136–0.579	0.001*
**Adjuvant chemotherapy**						
No vs. yes	0.847	0.455–1.538	0.566			
**Adjuvant radiotherapy**						
No vs. yes	0.049	0.000–3995.780	0.601			
**CD8 + cytotoxic T cells expression (tumor)**						
Low vs. high	2.995	1.563–5.738	0.001*	1.144	0.516–2.535	0.741
**CD8 + cytotoxic T cells expression (peritumor)**						
Low vs. high	0.769	0.425–1.392	0.385			
**CD15 + neutrophils expression (tumor)**						
Low vs. high	0.701	0.38–1.270	0.241			
**CD15 + neutrophils expression (peritumor)**						
Low vs. high	0.486	0.263–0.898	0.021*	0.611	0.324–1.154	0.129
**SMAD4 expression**						
Low vs. high	3.971	2.179–7.236	<0.0001*	4.329 *	1.974–9.495	<0.0001*

*Means statistically significant.

### Relationship between CD8+ cytotoxic T cells and CD15+ neutrophils with clinicopathologic features and survival of patients with HPC

**FIGURE 3:**
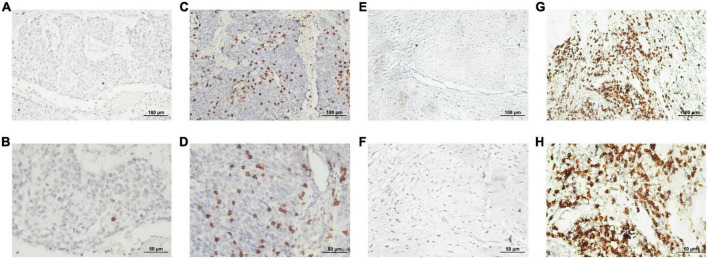
Representative photomicrographs of immunohistochemical staining for CD8 + cytotoxic T cells. **(A–D)** Low and high intratumoral infiltration of CD8 + cytotoxic T cells, respectively. **(E–H)** Low and high peritumoral infiltration of CD8 + cytotoxic T cells, respectively (upper panels, ×200 magnification; lower panels, ×400 magnification).

The staining density of CD8 + cytotoxic T cells was manually calculated under a microscope. The median counts of CD8 + cytotoxic T cells were 21.7 in HPC tissues and 109.4 in peritumor tissues. High CD8 + cytotoxic T cell level was defined as an increased number of positively stained cells compared to the median count; the opposite results were considered as low level. Representative images are shown in Figure 3, and the relationship between intratumoral CD8 + cytotoxic T cells and clinical parameters is depicted in Table 4. Next, OS and DFS with respect to CD8 + cytotoxic T cells in both tumor and peritumor tissues were analyzed. Interestingly, infiltration of CD8 + cytotoxic T cells was associated with a better OS (Figure 2C) and DFS (Figure 2D) in HPC tumor tissues. However, the density of CD8 + cytotoxic T cells in peritumor (Figures 2E, F) tissues was not significant. The results of univariate analysis were similar; however, multivariate analysis did not suggest that CD8 + cytotoxic T cell expression affected OS and DFS (Tables 2, 3).

**TABLE 4:** The relationship between intratumoral CD8 + cytotoxic T cell infiltration and clinical characteristics.

Variables	High CD8 + cytotoxic T cell expression (*n* = 48)	Low CD8 + cytotoxic T cell Expression (*n* = 49)	*P*-value
**Age (year)**			
<59	29	18	0.0196*
≥59	19	31	
**Gender**			
Male	46	48	0.6171
Female	2	1	
**TNM stage**			
I + II	0	1	>0.9999
III + IV	48	48	
**Tumor differentiation**			
Well differentiated	37	43	0.1669
Poor differentiated	11	6	
**Surgical margin**			
Positive	4	4	>0.9999
Negative	44	45	
**Vascular invasion**			
Yes	9	10	0.837
No	39	39	
**Adjuvant chemotherapy**			
Yes	26	32	0.2633
No	22	17	
**Adjuvant radiotherapy**			
Yes	0	1	>0.9999
No	48	48	
**T stage**			
1–2	9	14	0.2555
3–4	39	35	
**N stage**			
0–1	20	15	0.2570
2–3	28	34	

*Means statistically significant.

**FIGURE 4:**
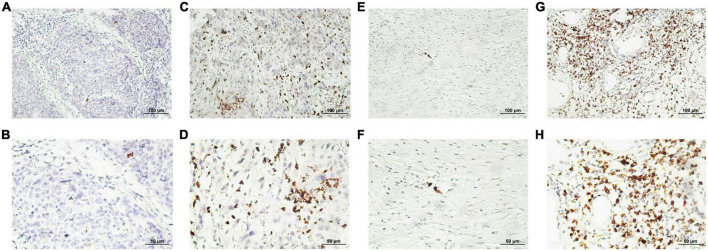
Representative photomicrographs of immunohistochemical staining of CD15 + neutrophils. **(A–D)** Low and high intratumoral levels of CD15 + neutrophils, respectively. **(E–H)** Low and high peritumoral levels of CD15 + neutrophils, respectively (upper panels, ×200 magnification; lower panels, ×400 magnification).

The calculation and assessment of CD15 + neutrophils were performed using similar methods as described for CD8 + cytotoxic T cells. The median counts of CD15 + neutrophils were 7.4 in HPC tissues and 44.6 in peritumor tissues. Representative images are shown in Figure 4, and the relationship between peritumoral CD15 + neutrophil level and clinical features is depicted in Table 5. The survival curves revealed that increased CD15 + neutrophils in the peritumor tissues were associated with poor OS (Figure 2I) and DFS (Figure 2J), although the number of CD15 + neutrophils did not significantly affect OS and DFS in the tumor tissues of HPC (Figures 2G, H). Univariate analysis confirmed that peritumoral CD15 + neutrophils impacted OS and DFS, and the results of multivariate analysis were almost significant (Tables 2, 3).

**TABLE 5:** The relationship between peritumoral CD15 + neutrophil level and clinical characteristics.

Variables	High CD15 + neutrophil expression (*n* = 48)	Low CD15 + neutrophil expression (*n* = 49)	*P* value
**Age (year)**			
<59	27	20	0.1283
≥59	21	29	
**Gender**			
Male	48	46	0.2423
Female	0	3	
**TNM stage**			
I + II	1	0	0.4948
III + IV	47	49	
**Tumor differentiation**			
Well differentiated	40	40	0.8257
Poor differentiated	8	9	
**Surgical Margin**			
Positive	5	3	0.4865
Negative	43	46	
**Vascular invasion**			
Yes	7	12	0.219
No	41	37	
**Adjuvant chemotherapy**			
Yes	29	29	0.9015
No	19	20	
**Adjuvant radiotherapy**			
Yes	1	0	0.4948
No	47	49	
**T stage**			
1–2	11	12	0.8555
3–4	37	37	
**N stage**			
0–1	18	17	0.7736
2–3	30	32	

In summary, a high density of CD8 + cytotoxic T cells in HPC tumor tissues, rather than in peritumor tissues, suggested a favorable prognosis in patients with HPC, and excessive CD15 + neutrophils in the peritumor promoted unfavorable clinical outcomes.

### Relation between SMAD4 and CD8 + cytotoxic T cells/CD15 + neutrophils in both tumor and peritumor tissues

**FIGURE 5:**
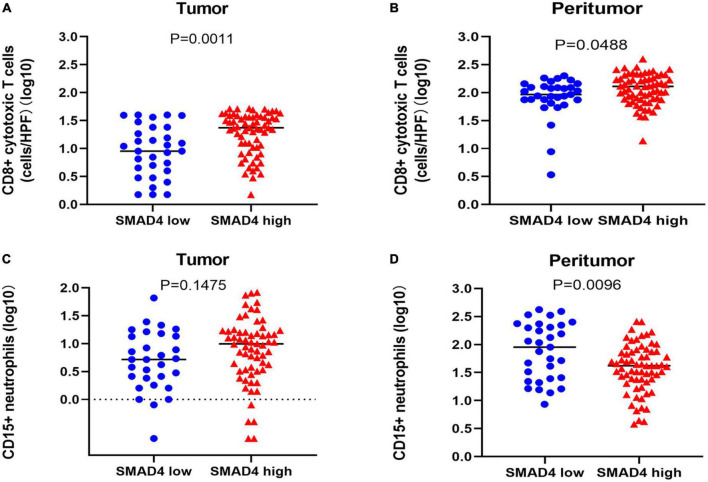
Correlation between SMAD4 status and CD8 + cytotoxic T cell and CD15 + neutrophil counts in HPC. SMAD4 expression level proportionally correlated with CD8 + cytotoxic T cell counts in tumor **(A)** and peritumor **(B)** tissues. SMAD4 expression level was inversely related to CD15 + neutrophils in peritumor areas **(D)** but not in tumor areas **(C)**. HPC, hypopharyngeal carcinoma.

To explore whether SMAD4 affects the immune responses of CD8 + cytotoxic T cells/CD15 + neutrophils, we used the Mann–Whitney U test. Infiltration of CD8 + cytotoxic T cells paralleled SMAD4 expression in both the tumor (Figure 5A) and peritumor (Figure 5B); the results were more apparent in the tumor tissues. The total number of CD15 + neutrophils in the peritumor (Figure 5D) was inversely correlated with SMAD4 expression level, which was not observed in tumor tissues (Figure 5C).

**FIGURE 6:**
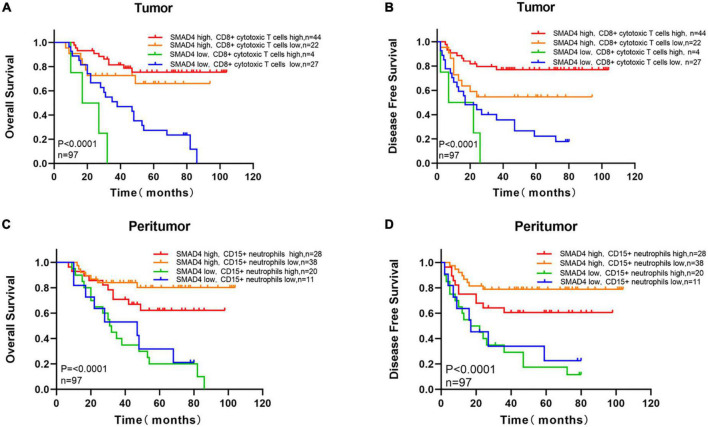
Survival analysis with respect to SMAD4 expression combined with infiltration of intratumoral CD8 + cytotoxic T cells and density of peritumoral CD15 + neutrophils. Overall survival **(A)** and disease-free survival **(B)** with respect to high/low SMAD4 expression levels with high/low infiltration levels of CD8 + cytotoxic T cells tumor infiltration. Overall survival **(C)** and disease-free survival **(D)** with respect to high/low SMAD4 expression levels with high/low density of CD15 + neutrophils in peritumor area.

We next investigated the co-effect of SMAD4 expression and intratumoral CD8 + cytotoxic T cell infiltration on the prognosis of HPC. The following four groups were defined: high SMAD4 expression/high CD8 + cytotoxic T cell infiltration group, high SMAD4 expression/low CD8 + cytotoxic T cell infiltration group, low SMAD4 expression/high CD8 + cytotoxic T cell infiltration group, and low SMAD4 expression/low CD8 + cytotoxic T cell infiltration group. As expected, increased SMAD4 expression level with abundant intratumoral CD8 + cytotoxic T cell infiltration were associated with the best OS and DFS (Figures 6A, B). The conflation of high SMAD4 expression level and lower CD8 + cytotoxic T cell infiltration also indicated a positive prognostic value. The poor clinical outcome was observed when both SMAD4 expression level and CD8 + cytotoxic T cell counts were below the median value.

To predict the interactions of SMAD4 and CD15 + neutrophils, we applied the semblable method. OS and DFS were highest when SMAD4 was abundantly expressed and fewer CD15 + neutrophils were present in the peritumor (Figures 6C, D). Shorter OS and DFS were observed when SMAD4 was lower and CD15 + neutrophil greatly accumulated.

Overall, SMAD4 and CD8 + cytotoxic T cells interacted in parallel and appeared to have synergistic effects in tumors, predicting a considerably improved prognosis. Clinical outcomes worsened when the CD15 + neutrophil levels were high in SMAD4-deficient peritumor. Thus, SMAD4 affects survival via mediating CD8 + cytotoxic T cell- and CD15 + neutrophil-regulated immune response in HPC.

## Discussion

HPC is typically diagnosed in advanced stages, and currently available treatments have limitations, compromising the patient’s quality of life. Hence, a useful biomarker for identifying HPC is needed.

Elevated levels of SMAD4 or CD8 + cytotoxic T cells are associated with a longer life span, whereas increased CD15 + neutrophil level indicates decreased survival in various cancers. As a suppressor gene, SMAD4 plays a key role in the TGF-β signaling pathway by regulating cell-cycle arrest and apoptosis. SMAD4 functions at the G1/S checkpoint and causes cells to stall in G1 phase, resulting in cell-cycle arrest (21). The DNA damage and genomic instability caused by SMAD4-deletion results in poor survival and drug resistance in patients with squamous cell carcinoma of the head and neck (22–24). Accumulating evidence has revealed that patients with loss of SMAD4 have more chance to develop metastatic relapse (25, 26). The underlying mechanism perhaps related to TGFβ1-induced autophagy relying on expression of SMAD4. In SMAD4-negative PDAC cells, TGFB1-induced autophagy triggers the expression of MAPK/ERK, giving rise to migration rather than proliferation, which accounts for metastasis (27). We further analyzed relation between SMAD4 and local recurrence as well as distant metastasis. Our data shows that the expression of SMAD4 correlated to local recurrence and distant metastasis and the latter is more statistically significant. SMAD4 sufficiency in HPC is less likely to have metastasis compared with SMAD4 depletion.

CD8 + cytotoxic T cells are considered as the tumor-infiltrating lymphocytes subset because of their ability to directly kill cancer cells, serving as an anti-tumor effector in immune responses (28, 29). Neutrophil, abundant in human peripheral blood(50%-70%) (30) is the first line of defense against infection. Meanwhile, it also takes up higher percentages in peritumoral region rather than tumor site, which plays a crucial role by impairing antitumor immunity (31).Tumor-associated neutrophils have two types of subpopulations: anti-tumoral(N1) and pro-tumoral(N2) and CD15 + neutrophils are inclined to present a pro-tumoral phenotype in peritumoral areas among tumor-associated neutrophils, promoting tumor initiation and promotion (32) and leading to shorter survival (33, 34).

In this study, we consolidated the prognostic values of SMAD4, CD8 + cytotoxic T cells, and CD15 + neutrophils; specifically, we assessed their combination effect and correlation. SMAD4 expression was inversely related to age, explaining why SMAD4 deficiency commonly occurs in the elderly and why cancers tend to occur in senile people. Kaplan–Meier analysis and univariate and multivariate analysis clearly showed that high levels of SMAD4 were associated with improved OS and DFS. Additionally, SMAD4 was positively related to intratumoral CD8 + cytotoxic T cells and negatively related to peritumoral CD15 + neutrophils. To further investigate our hypothesis, we compared OS and DFS in various conditions. Our data supported that elevated level of both SMAD4 and intratumoral CD8 + cytotoxic T cells synergistically inhibit recurrence and distant metastasis in HPC, resulting in the best OS and DFS. A low level of SMAD4 and high levels of CD15 + neutrophils in para-cancerous tissues were associated with the shortest OS and DFS. These results suggest that SMAD4 is an indispensable element in the immune environment, affecting the quantitative distribution of immune cells and determining the prognostic value by mediating CD8 + cytotoxic T cells, CD15 + neutrophils, and immune responses in HPC.

These results agree with those of previous studies highlighting that SMAD4 participates in immune responses. Kim et al. suggested that Smad4-dependent signaling in T cells makes decreases the susceptibility to spontaneous gastrointestinal tumorigenesis by maintaining homeostasis, indicating that SMAD4 signaling in T cells is required to suppress gastrointestinal cancer (35). Furthermore, Principe et al. suggested that SMAD4-intact increased T cell infiltration by recruiting more T cells and therefore SMAD4 is an indicator of a survival advantage in patients with pancreatic ductal carcinoma (36). SMAD4 is required for the differentiation of CD8 + T cells during inflammatory response (37, 38). Ogawa et al. found that neutrophil infiltration into the peritumoral stroma was higher in SMAD4-negative colorectal cancer, and loss of SMAD4 promoted colorectal cancer progression (39). These findings suggest that SMAD4 is an essential component in immunoregulation either by mediating T lymphocyte immune activity or neutrophil immune responses. We hypothesize SMAD4 interacts with immune cells through some specific cytokines, such as chemokine (C-X-C motif) ligand 1 (CXCL1) and CCL15. An et al. reckoned SMAD4-deficient gastric cancer inhibit dendritic cells(DC)differentiation and subsequently cytotoxic T cells infiltration via CXCL1 (40). Yamamoto et al. viewed a lack of SMAD4 in colorectal cancer cells facilitates recruitment of neutrophils (CD15 + included) by releasing CCL15, which contributes to lung metastasis (41).

Despite these analogical insights, other studies showed conflicting results. Recently, Xiong et al. demonstrated that the absence of SMAD4 promotes tumor cell immunogenicity and may trigger activation of subsequent CD8 + T cells for tumor control (42). In addition, according to Li et al., SMAD4-depletion significantly augmented anti-tumor immunity (43). These seemingly discrepant observations suggest that the relationship between SMAD4 and tumor immune activity relies on the anatomical structure, histological type, and multiple functions of SMAD4 during different cancer stages.

Treating hypopharyngeal carcinoma is difficult, and immunotherapy (e.g., treatment with immune checkpoint inhibitors) has led to new treatment options for solid tumors. Our findings support that SMAD4 is an immune promoter that enhances the ability of CD8 + cytotoxic T cells to suppress tumors and impair CD15 + neutrophils from accelerating malignant progression in HPC. To our knowledge, this is the first study to identify the relationship between SMAD4 and immune responses in HPC. SMAD4 is a promising candidate biomarker for predicting HPC prognosis and may be a immunomodulator in the HPC microenvironment.

Although our study provides insight on the role and combined prognostic value of SMAD4, CD8 + cytotoxic T cells, and CD15 + neutrophils, there were several limitations to our study. First, because of technical limitations, some pathological specimens could not be obtained for analysis, leading to a small sample size. Additionally, other cytokines or modulatory molecules may be involved in this process but have not been identified. Experiments *in vitro* or *in vivo* are needed to verify the mechanism by which SMAD4 impacts the levels of CD8 + cytotoxic T cells and CD15 + neutrophils.

## Conclusion

Our study revealed the role of SMAD4 and increased intratumoral infiltration of CD8 + cytotoxic T cells, and their combination, as an indicator of longer OS and DFS in HPC. In contrast, a combination of depletion of SMAD4 and a high density of peritumoral CD15 + neutrophils appears to confer worse survival. SMAD4 is a promising predictive biomarker in HPC and may be useful as a future target in immunotherapy.

## Data availability statement

The original contributions presented in this study are included in the article/supplementary material, further inquiries can be directed to the corresponding authors.

## Ethics statement

This study was approved by the Ethics Committee of Sun Yat-sen Memorial Hospital, which granted a waiver of informed consent to use patient records and pathological samples for research.

## Author contributions

CLC and YML: study concepts and design and quality control of data and algorithms. JJS: data acquisition and manuscript editing. JJS and JLW: data analysis and interpretation and statistical analysis. JXD and YXL: manuscript preparation. JLW, CLC, YML, JXD, and YXL: manuscript review. All authors contributed to the article and approved the submitted version.
